# Insulin growth factor axis and cardio-renal risk in diabetic kidney disease: an analysis from the CREDENCE trial

**DOI:** 10.1186/s12933-023-01916-2

**Published:** 2023-07-12

**Authors:** Reza Mohebi, Yuxi Liu, Michael K. Hansen, Yshai Yavin, Naveed Sattar, Carol A. Pollock, Javed Butler, Meg Jardine, Serge Masson, Hiddo J. L. Heerspink, James L. Januzzi Jr

**Affiliations:** 1 Cardiology Division, Massachusetts General Hospital and Harvard Medical School, 55 Fruit Street, Boston, MA 02114 USA; 2 Janssen Research Development, LLC, Spring House, Montgomery, PA USA; 3 BHF Glasgow Cardiovascular Research Centre, University of Glasgow, Glasgow, UK; 4 Kolling Institute, Royal North Shore Hospital University of Sydney, Sydney, NSW Australia; 5 University of Mississippi Medical Center, Jackson, MS USA; 6 Baylor Scott & White Institute, Dallas, TX USA; 7 The George Institute for Global Health, UNSW Sydney, Sydney, NSW Australia; 8 NHMRC Clinical Trials Centre, University of Sydney, Sydney, NSW Australia; 9 Concord Repatriation General Hospital, Sydney, NSW Australia; 10 Roche Diagnostics International, Rotkreuz, Switzerland; 11 Department Clinical Pharmacy and Pharmacology, University of Groningen, Groningen, The Netherlands; 12 Heart Failure and Biomarker Trials, Baim Institute for Clinical Research, Boston, MA USA

**Keywords:** IGF-1, Diabetes mellitus, Chronic kidney disease, Canagliflozin

## Abstract

**Background:**

The insulin-like growth factors (IGF) play a crucial role in regulating cellular proliferation, apoptosis, and key metabolic pathways. The ratio of IGF-1 to IGF binding protein-3 (IGFBP-3) is an important factor in determining IGF-1 bioactivity. We sought to investigate the association of IGF-1 and IGFBP-3 with cardio-renal outcomes among persons with type 2 diabetes.

**Methods:**

Samples were available from 2627 individuals with type 2 diabetes and chronic kidney disease that were randomized to receive canagliflozin or placebo and were followed up for incident cardio-renal events. Primary outcome was defined as a composite of end-stage kidney disease, doubling of the serum creatinine level, or renal/cardiovascular death. IGF-1 and IGFBP-3 were measured at baseline, Year-1 and Year-3. Elevated IGF-1 level was defined according to age-specific cutoffs. Cox proportional hazard regression was used to investigate the association between IGF-1 level, IGFBP-3, and the ratio of IGF-1/IGFBP-3 with clinical outcomes.

**Results:**

Elevated IGF-1 was associated with lower glomerular filtration rate at baseline. Treatment with canagliflozin did not significantly change IGF-1 and IGFBP-3 concentrations by 3 years (p-value > 0.05). In multivariable models, elevated IGF-1 (above vs below age-specific cutoffs) was associated with the primary composite outcome (incidence rate:17.8% vs. 12.7% with a hazard ratio [HR]: 1.52; 95% confidence interval CI 1.09–2.13;*P*: 0.01), renal composite outcome (HR: 1.65; 95% CI 1.14–2.41; *P*: 0.01), and all-cause mortality (HR: 1.52; 95% CI 1.00–2.32; *P*; 0.05). Elevations in log IGFBP-3 did not associate with any clinical outcomes. Increase in log IGF-1/IGFBP-3 ratio was also associated with a higher risk of the primary composite outcome (HR per unit increase: 1.57; 95% CI 1.09–2.26; *P*; 0.01).

**Conclusions:**

These results further suggest potential importance of IGF biology in the risk for cardio-renal outcomes in type 2 diabetes. SGLT2 inhibition has no impact on the biology of IGF despite its significant influence on outcomes.

*Trial registration*: CREDENCE; ClinicalTrials.gov Identifier: NCT02065791.

**Supplementary Information:**

The online version contains supplementary material available at 10.1186/s12933-023-01916-2.

## Background

Individuals with type 2 diabetes mellitus are at increased risk of cardio-renal complications. Within the altered hormonal milieu among those with type 2 diabetes are changes in the insulin growth factor axis; recent studies have examined the role of insulin growth factor-1 (IGF-1) in the risk for complications from type 2 diabetes [1]. IGF-1 is a 70-amino acid peptide, structurally homologous to pro-insulin, synthesized mainly in the liver upon growth hormone (GH) stimulation [2]. Molecular studies have revealed that IGF-1 promotes cellular growth, inhibits cell apoptosis, stimulates glucose uptake by muscle and heart cells, and enhances glycogen, lipid, and protein synthesis [3, 4]. These metabolic effects are regulated by a complex interaction between GH, insulin, IGF-1, and 6 soluble high-affinity IGF-binding proteins [5].

Abnormal concentrations of IGF-1 are linked with obesity [6], metabolic syndrome [7], type 2 diabetes [8], atherosclerosis [9], heart failure (HF) [10, 11], and diabetic kidney disease (DKD) [12]. Furthermore, recent data have implicated insulin-like growth factor binding proteins (IGFBP) in cardio-renal risk in those with and without type 2 diabetes [13]. The most abundant peptide in the IGFBP family is IGFBP-3 [14]. It has a high affinity for IGF-1 and alters the interaction between IGF-1 and IGF-1 receptor. The ratio of IGF-1 to IGFBP-3 is considered as a parameter of IGF-1 bioactivity [15].

Further research is needed to determine the significance of IGF-1, IGFBP-3, and the ratio between the two in assessing cardio-renal risk, as previous studies have yielded conflicting results regarding their association with adverse clinical outcomes [16]. Furthermore, an understanding of how therapies with benefits on cardio-renal risk in diabetes intersect with concentrations of these peptides is unknown. Accordingly, in the present analysis, using data from CREDENCE trial (Canagliflozin and Renal Events in Diabetes with Established Nephropathy Clinical Evaluation; ClinicalTrials.gov Identifier: NCT02065791), we sought to investigate the association of IGF-1, IGFBP-3 and IGF-1/IGFBP-3 ratio with incident cardio-renal outcomes and evaluated effect of canagliflozin on their concentrations.

## Methods

### Study design and patient population

The trial design, baseline patient characteristics, and the main study results from the CREDENCE trial have been published previously [17, 18]. Briefly, CREDENCE was a placebo-controlled trial of canagliflozin 100 mg versus placebo in 4401 persons with type 2 diabetes and DKD. Study participants had a minimum glycated hemoglobin between 6.5% and 12.0% and were required to have an estimated glomerular filtration rate (eGFR) between 30 and 90 mL/min/1.73 m^2^ and urine albumin creatinine ratio (UACR) > 300 to 5000 mg/g. All subjects had to be treated with angiotensin converting enzyme inhibitor(ACEi) or angiotensin receptor blocker (ARB) at randomization.

In this analysis, only those study participants with available plasma for analysis of IGF-1 and IGFBP-3 at baseline were included (N = 2627). Plasma samples were collected at baseline, 1 year, and 3 years, and stored at – 80 ℃ degrees centigrade. IGF-1 was measured using an automated electrochemiluminescence immunoassay (Roche Diagnostics, Mannheim, Germany). This method is standardized against the WHO International Standard 02/254. Detection limit was 7 ng/mL and coefficient of variation for repeatability was ≤ 3.5%

There were 4 main goals of this analysis. First, we determined the distribution of biomarkers at baseline. Second, we evaluated canagliflozin’s effect on biomarker concentrations from baseline to 1 year and baseline to 3 years. Third, we evaluated the association between biomarker concentrations at baseline (or their change from baseline to Year 1) and cardiovascular (CV) and kidney outcomes. Clinical endpoints examined included the primary composite endpoint of CREDENCE (a composite of end-stage kidney disease, doubling of the serum creatinine level, or renal/CV death), the renal composite endpoint (a composite of end-stage kidney disease, doubling of the serum creatinine level, or renal death), as well as the composite of heart failure (HF) hospitalization or CV death, HF hospitalization, all-cause death, and CV death. Fourth, we evaluated the effect of canagliflozin on risk as a function of concentrations of IGF-1, IGFBP-3, or their ratio.

### Statistical analysis

Biomarkers were log transformed because of their skewed distribution. Median (interquartile) and count (frequency) were used to present continuous and categorical variables. Kruskal–Wallis, ANOVA, and chi-square tests were used to compare the baseline characteristics of study population across IGF-1 quartiles, IGFBP-3, and IGF-1/IGFBP-3 ratio as appropriate. To evaluate the effect of canagliflozin on biomarker concentrations, comparisons of geometric mean (95% CI) concentrations were performed in Years 1 and 3. For change from baseline to Year 1, a base linear model was constructed for each log-transformed biomarker at Year 1 by selecting important baseline covariates in patients randomized to placebo in the main study based on Bayesian Information Criterion. The candidate covariates at baseline included continuous variables: age, eGFR, body mass index, systolic blood pressure, hemoglobin A1c, duration of diabetes mellitus, UACR, log transformed NTproBNP and categorical variables: history of HF, and history of diuretic treatment. Cox proportional hazard regression was implemented to assess the association between biomarker concentrations with clinical outcomes, including treatment and treatment-by-biomarker interaction in the models with selected covariates. To do so, log-transformed concentrations of IGF-1 and IGFBP-3 or their ratio were evaluated with hazard ratio (HR) and 95% CI expressed per 1-unit change in each measure. Additionally, dichotomous cutoffs for IGF-1 based on age were also applied ^19^.

All hypotheses were 2-sided, with a p-value < 0.05 considered statistically significant. All statistical analyses were performed using the R version 4.2.2 (R Foundation for Statistical Computing, Vienna, Austria. URL: https://www.R-project.org/).

## Results

Additional file 1: Figure S1 details the study flow for the present analysis. The baseline study sample consisted of 2627 individuals with diabetic kidney disease**.**

Table 1 details the baseline characteristics of the study population across IGF-1 quartile groups. Patients with the highest quartile were younger, more likely to be male and Black, had a lower prevalence of coronary artery disease, lower eGFR level, lower systolic blood pressure, higher diastolic blood pressure, and lower diabetes mellitus duration compared to other quartiles. Baseline characteristics according to IGFBP-3 and IGF-1/IGFBP-3 ratio quartiles are detailed in Additional file 1: Tables S1, S2.

**Table 1:** Baseline characteristics of study population stratified by IGF-1 quartile

	Q1 (N = 643)	Q2 (N = 642)	Q3 (N = 642)	Q4 (N = 642)	*P*
IGF-1, ng/mL	62 (51, 70)	92 (84, 98)	119 (112, 128)	165 (149, 190)	< 0.001
Canagliflozin, n (%)	306 (47.6)	320 (49.8)	307 (47.8)	340 (53.0)	0.19
Age, years, mean (SD)	66.32 (8.29)	64.51 (8.66)	62.70 (8.97)	59.56 (8.90)	< 0.001
Male, n (%)	374 (58.3)	424 (66.5)	417 (65.5)	487 (75.9)	< 0.001
Race, n (%)					0.02
White	492 (76.6)	467 (73.2)	451 (70.8)	424 (66.0)	
Asian	69 (10.7)	84 (13.2)	81 (12.7)	96 (15.0)	
Black	21 (3.3)	32 (5.0)	40 (6.3)	47 (7.3)	
Comorbidities, n (%)					
Heart failure	92 (14.3)	85 (13.2)	90 (14.0)	64 (10.0)	0.08
Smoking	89 (13.8)	96 (15.0)	89 (13.9)	106 (16.5)	0.49
Hypertension	623 (96.9)	627 (97.7)	621 (96.7)	611 (95.2)	0.09
Coronary disease	192 (29.9)	195 (30.4)	192 (29.9)	149 (23.2)	0.01
Cerebrovascular disease	99 (15.4)	101 (15.7)	97 (15.1)	87 (13.6)	0.70
Peripheral artery disease	172 (26.7)	143 (22.3)	168 (26.2)	160 (24.9)	0.26
Chronic kidney disease	595 (94.9)	596 (94.9)	609 (96.4)	601 (95.4)	0.56
Obesity	384 (59.8)	357 (56.0)	380 (59.3)	353 (55.0)	0.22
eGFR, mL/min/1.73 m^2^ mean (SD)	58.81 (18.26)	57.54 (18.16)	56.05 (18.16)	54.46 (18.16)	< 0.001
Body mass index, kg/m^2^ mean (SD)	32.32 (6.67)	31.66 (6.01)	31.91 (6.27)	31.51 (6.07)	0.10
SBP, mmHg mean (SD)	141.92 (16.00)	140.80 (15.75)	138.08 (15.33)	139.92 (15.80)	< 0.001
DBP, mmHg mean (SD)	76.75 (9.24)	78.10 (9.28)	77.07 (9.69)	79.39 (9.45)	< 0.001
Hemoglobin A1c, mmol/mol mean (SD)	67 (14.5)	66 (14.1)	67 (14.9)	66 (13.6)	0.39
LDL-C, mmol/L Median (IQR)	2.25 (1.66, 2.97)	2.30 (1.68, 3.00)	2.20 (1.66, 3.18)	2.35 (1.76, 3.13)	0.20
HDL-C, mmol/L Median (IQR)	1.11 (0.91, 1.32)	1.11 (0.93, 1.37)	1.09 (0.91, 1.34)	1.09 (0.93, 1.29)	0.39
Triglycerides, mmol/L Median (IQR)	1.83 (1.32, 2.62)	1.80 (1.31, 2.80)	1.80 (1.28, 2.59)	1.83 (1.34, 2.54)	0.86
Diabetes duration, years, mean (SD)	17.7 (9.3)	16.0 (8.7)	15.8 (8.5)	14.5 (8.0)	< 0.001
Albumin creatinine ratio, median (IQR)mg/g, median (IQR)	107 (55, 192)	105 (51, 203)	98 (50, 193)	101 (55, 202)	0.73
Medications, n (%)					
Diuretic use	349 (54.3)	303 (47.2)	311 (48.4)	302 (47.0)	0.03
Statin use	467 (72.6)	464 (72.3)	470 (73.2)	445 (69.3)	0.41
Antithrombotic use	433 (67.3)	407 (63.4)	420 (65.4)	349 (54.4)	< 0.001
Beta blocker	290 (45.1)	276 (43.0)	268 (41.7)	259 (40.3)	0.36
Metformin	390 (60.7)	387 (60.3)	384 (59.8)	393 (61.2)	0.96
GLP-1 receptor agonist	36 (5.6)	37 (5.8)	24 (3.7)	30 (4.7)	0.31
Insulin	456 (70.9)	422 (65.7)	424 (66.0)	407 (63.4)	0.03
Sulfonylureas	133 (20.7)	179 (27.9)	170 (26.5)	205 (31.9)	< 0.001
Biomarkers, median (IQR)					
NT-proBNP, pg/mL	239 (101, 569)	195 (94, 440)	172 (81, 458)	128 (63, 301)	< 0.001
Troponin T, ng/mL	18 (12, 26)	18 (12, 27)	19 (12, 29)	21 (14, 34)	< 0.001
IGFBP-3, ng/mL	2174 (1767, 2648)	2928 (2448, 3521)	3519 (2944, 4150)	4315 (3681, 5015)	< 0.001
IGF-1/IGFBP-3 × 1000 ratio	26.8 (21.5, 32.3)	31.8 (25.6, 37.7)	34.7 (28.9, 39.9)	41.0 (35.0, 47.3)	< 0.001

*IGF-1* insulin-like growth factor-1, *egfr* estimated glomerular filtration rate, *SBP* systolic blood pressure, *DBP* diastolic blood pressure, *LDL-C* low-density lipoprotein cholesterol, *HDL-C* high-density lipoprotein cholesterol, *GLP-1* glucagon-like peptide 1, *IQR* interquartile range, *NT-proBNP* N terminal pro B type natriuretic peptides, *IGFBP* insulin-like growth factor binding protein

Table 2 shows unadjusted geometric mean (95% CI) concentrations of biomarkers at baseline, Year 1, and Year 3. IGF-1, IGFBP-3 levels, and the IGF-1/IGFBP-3 ratio remained relatively constant during 3 years of follow-up among both canagliflozin and placebo groups. To explore treatment-related effect on biomarker concentrations, geometric mean ratio of Year 1/baseline was examined in adjusted analyses. In these adjusted models, treatment with canagliflozin did not significantly change concentrations of IGF-1 and IGFBP-3 over time (Additional file 1: Table S3).

**Table 2:** Unadjusted geometric mean (95% CI) concentrations of biomarkers at baseline, Year 1, and Year 3

Biomarker	Canagliflozin	Placebo	*P*
Baseline			
IGF-1, ng/L	101.20 (98.84, 103.62)	102.73 (100.22, 105.32)	0.20
IGFBP-3, ng/mL	3120.64 (3059.19, 3183.34)	3152.18 (3087.46, 3218.26)	0.68
IGF-1/IGFBP-3 × 1000	32.46 (31.92, 32.99)	32.59 (32.03, 33.17)	0.43
Year 1			
IGF-1, ng/L	105.91 (103.34, 108.55)	102.17 (99.52, 104.89)	0.08
IGFBP-3, ng/mL	3166.60 (3100.81, 3233.78)	3140.60 (3073.15, 3209.54)	0.42
IGF-1/IGFBP-3 × 1000	33.41 (32.85, 33.98)	32.53 (31.95, 33.12)	0.08
Year 3			
IGF-1, ng/L	101.80 (97.81, 105.95)	100.28 (96.12, 104.62)	0.89
IGFBP-3, ng/mL	3047.84 (2948.34, 3150.70)	3013.65 (2910.02, 3120.95)	0.62
IGF-1/IGFBP-3 × 1000	33.38 (32.53, 34.24)	33.33 (32.40, 34.28)	0.7

*CI* confidence interval, IGF-1: insulin-like growth factor-1, IGFBP: insulin growth factor binding protein

IGF-1 and IGFBP-3 were examined as continuous and dichotomous variables. To do so, elevated IGF-1 level is defined based on age-specific cutoffs, as the level tends to decrease significantly with age [19] (Additional file 1: Table S4). Patients with elevated IGF-1 were younger, were more likely to be male and Black, had lower eGFR at baseline, and had a longer duration of type 2 diabetes compared to patients with lower concentrations of the biomarker (Additional file 1: Table S5). Concentrations of IGF-1 and IGFBP-3 across chronic kidney disease (CKD) stages are detailed in Additional file 1: Table S6. Patients with stage 4 CKD had higher concentration of IGF-1 compared to other stages. IGFBP-3 concentrations were similar across CKD stages.

Additional file 1: Figure S2 depicts the association of continuous log IGF-1, IGFBP-3, and IGF-1/IGFBP-3 ratio with primary composite outcome using restricted cubic spline modeling. Higher IGF-1 levels and IGF-1/IGFBP-3 ratio were associated with a higher risk of primary composite outcome. 

**Fig. 1:**
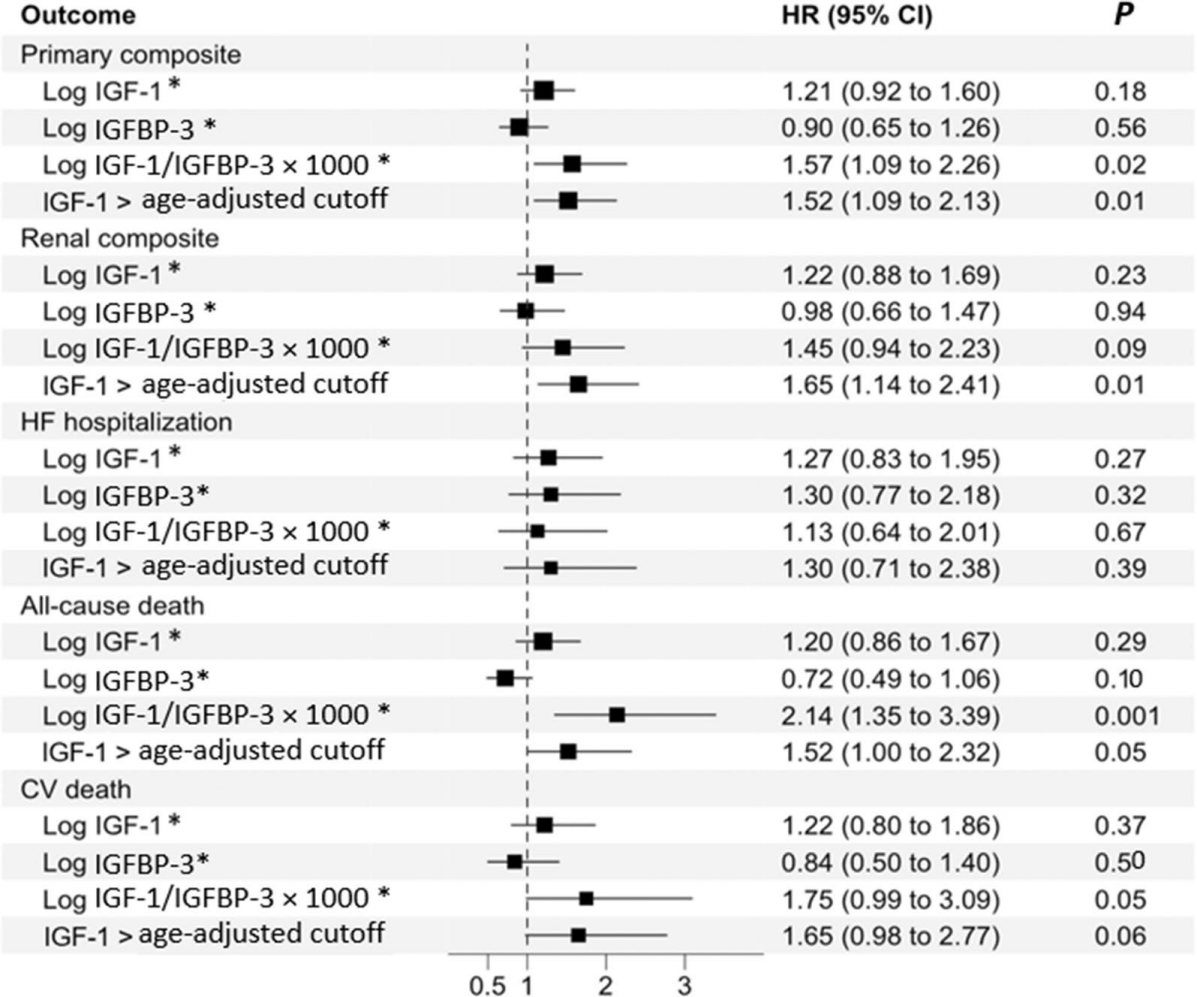
Association of IGF-1 and IGFBP-3 with clinical outcomes. In a multivariable-adjusted model, a 1-unit increase in log IGF1/IGFBP-3 ratio and elevated IGF-1 levels according to age-adjusted cutoff increased the risk of primary composite outcome, renal composite outcome, all-cause death, and CV death. *Per 1-unit increment. *HR* hazard ratio, *CI* confidence interval, *IGF-1* insulin-like growth factor-1, *IGFBP* insulin growth factor binding protein. *HF* heart failure, *CV* cardiovascular

**Fig. 2:**
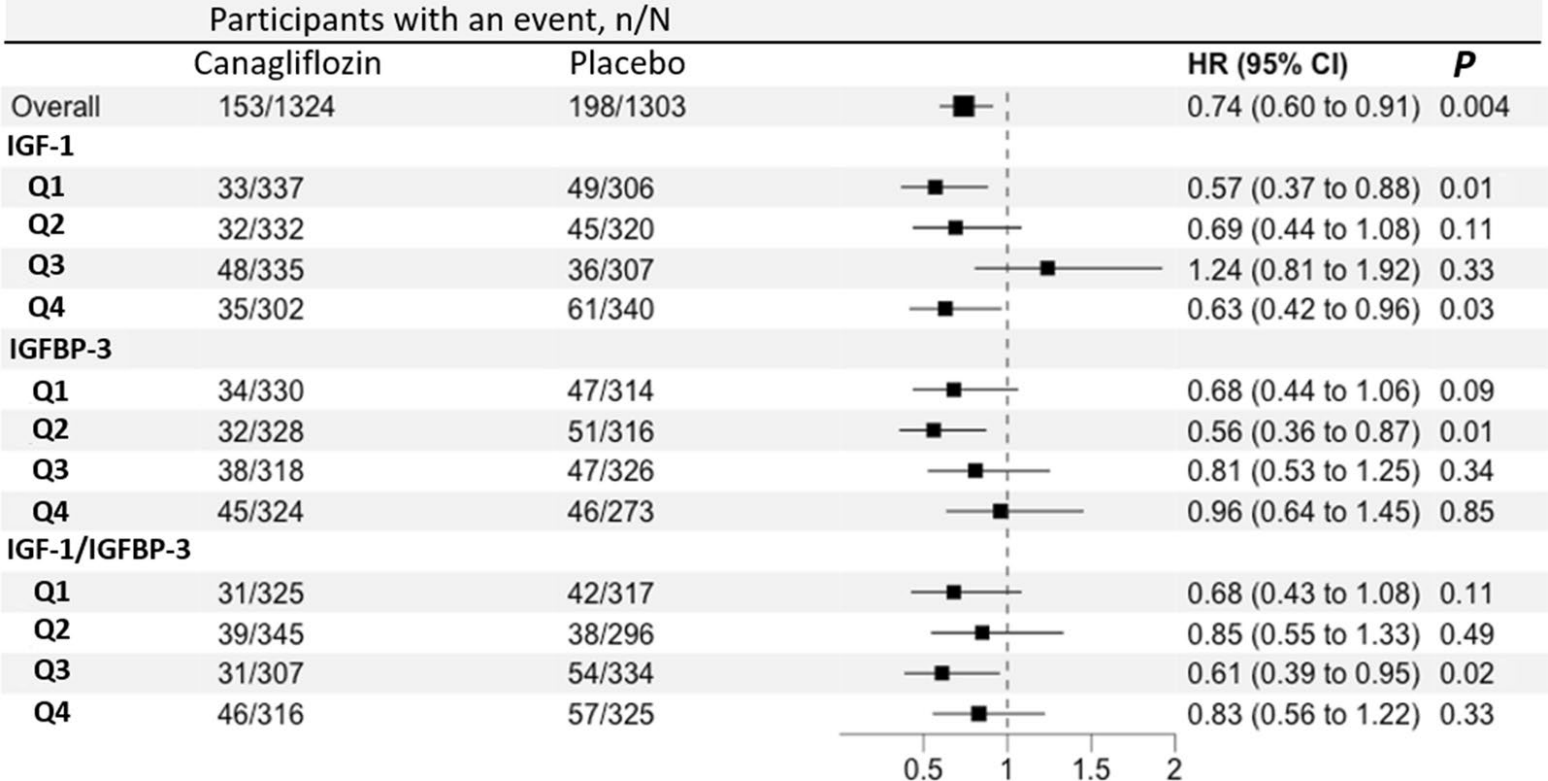
Efficacy of canagliflozin on lowering risk of primary composite outcome across IGF-1, IGFBP-3, and IGF-1/IGFBP-3 ratio quartiles. Effect of canagliflozin across quartiles of IGF-1, IGFBP-3, or their ratio was largely consistent relative to study outcomes. *IGF* insulin-like growth factor, *IGFBP* insulin growth factor binding protein, *HR* hazard ratio, *CI* confidence interval

Figure 1 demonstrates the association of continuous and dichotomous IGF-1, IGFBP-3, and IGF-1/IGFBP-3 with clinical outcomes. In the multivariable-adjusted model, 1-unit increase in log IGF-1 and IGFBP-3 was not associated with clinical outcomes (p-value > 0.1). However, elevated IGF-1 according to the age-specific cutoff was associated with the primary composite outcome (HR: 1.52, 95% CI 1.09–2.13, *P*: 0.01), renal composite outcome (HR: 1.65, 95% CI 1.14–2.41, *P*: 0.01) and all-cause mortality (HR: 1.52, 95%CI 1.00–2.32, *P*; 0.05). Also, an increase in the IGF-1/IGFBP-3 ratio was associated with primary, renal, CV death, and all-cause mortality outcomes (p values < 0.05). No treatment-by-biomarker interaction was present; thus, the effect of canagliflozin across quartiles of IGF-1, IGFBP-3, or their ratio was largely consistent relative to study outcomes (Fig. 2).

## Discussion

In this trial of patients with type 2 diabetes and CKD who were randomized to receive canagliflozin or placebo, we showed that baseline IGF-1 levels and IGF-1/IGFBP-3 ratio (but not IGFBP-3 concentrations) were associated with cardio-renal outcomes. Higher IGF-1 levels (for a given age) were associated with a greater risk of developing renal and all-cause mortality events. 3 years of therapy with canagliflozin did not significantly change IGF-1 and IGFBP-3 concentrations. Lastly, the benefits of canagliflozin to reduce cardio-renal events in this high-risk population were consistent across IGF-1 and IGFBP-3 strata. These findings provide evidence regarding the role of the IGF axis in risk for cardio-renal disease.

IGF-1 is an anabolic hormone that regulates cellular proliferation, apoptosis, and several metabolic pathways in the human body. Nearly all 98% of IGF-1 is bound to 1 of 6 IGFBPs in circulation. Owing to its longer half-life, IGFBP-3 is the most abundant member of the IGFBP family and accounts for 80% of all IGFBPs [20]; it binds to IGF-1 with high affinity and blocks IGF-1 access to the IGF-1 receptor. IGF-1 plays an intermediate role in glucose metabolism. Unlike GH, IGF-1 has a hypoglycemic effect by suppressing hepatic gluconeogenesis and stimulating peripheral glucose uptake [3]. IGF-1 levels increase as insulin resistance develops; with worsening of insulin resistance, the IGF-1 concentration reaches a plateau level, and subsequently, when glucose levels reach concentrations typical of type 2 diabetes, IGF-1 levels tend to decline [8, 21, 22]. Although the IGF-1/IGFBP-3 ratio is proposed as an index of IGF-1 bioavailability [23], it is essential to recognize other IGFBPs as they also modestly affect IGF actions [24].

While enhancing insulin sensitivity, growth-promoting properties of IGF-1 are proposed to play a role in developing complications of diabetes [1]. Emerging studies have shown the GH/IGF-1 axis alteration among patients with DKD [25] with IGF-1 production are thought to stimulate proliferation of renal mesenchymal cells and vascular proliferative changes [26]. Animal model studies have shown enhanced expression of renal IGF-1 receptor as a factor contributing to renal hypertrophy—a hallmark sign of DKD [27]. In addition, studies suggest that IGF-1 may have anti-natriuretic properties [4]. This is believed to occur through two mechanisms: firstly, it may directly affect the absorption of sodium by regulating the epithelial sodium channel in the distal nephron [28]. Secondly, IGF-1 overexpression may indirectly enhance the renin–angiotensin–aldosterone system. (RAAS) [29].

Despite the proposed mechanistic role of IGF-1 in DKD, results of clinical studies investigating the association between IGF-1 levels and kidney disease are inconclusive. In the NHANES study (National Health and Nutrition Examination Survey), Teppala and colleagues [30] showed that elevated IGF-1 levels were positively associated with CKD independent of conventional CKD risk factors. In line with this finding, Dittman and colleagues [31] demonstrated that increased serum IGF-1 levels were associated with decreased eGFR level. Contrary to these results, several studies have shown an association between low IGF-1 levels and adverse renal outcomes [32, 33]. In this study, we found a negative relationship between IGF-1 level and kidney function. One may assume that reduced renal excretion may play role in elevation IGF-1 concentration. However, this is possibly overly simplistic as elevated IGF activity may be linked with more severe kidney disease. Indeed, previous studies have established a connection between urinary excretion of insulin like growth factors and renal disease activity [34]; as a result, increased levels of IGF-1 in severe CKD patients may not be directly linked to renal excretion. Given biological rationale but mixed clinical studies, a rationale existed to examine the role of IGF-1 and IGFBP-3 in CREDENCE study participants.

Findings of our study corroborate studies indicating a detrimental association between elevated IGF-1 (when above age-specific cutoffs) as well as higher IGF-1/IGFBP-3 ratio on kidney function. As IGF-1 levels decrease by age, the findings from this study highlight the importance of considering IGF-1 age-specific cutoffs as well as incorporating the balance of IGF-1 bioactivity (reflected in the IGF-1/IGFBP-3 ratio) when studying IGF biology and cardio-renal risk. On the other hand, IGFBP-3 was not correlated with baseline kidney function and failed to predict any clinical events. IGFBP-3 has several IGF-1–dependent and IGF-1–independent functions [35]. The role of IGFBP-3 in type 2 diabetes and its complications requires further consideration.

The exact mechanism of the cardio-renal benefit of canagliflozin is still undetermined [36]. Beside lowering blood glucose, several mechanisms, including improved energy metabolism, vascular function, hemodynamic alterations, decreased oxidative stress, and reduction in inflammation, are proposed to mediate CV benefit of SGLT2 inhibitors. In this study, although higher IGF-1 levels were associated with renal outcomes, canagliflozin failed to lower IGF-1 levels and beneficial effects of canagliflozin in lowering adverse outcomes were observed across IGF-1 and IGFBP-3 levels. These findings imply that the beneficial effects of canagliflozin is likely independent of any changes in the IGF-1 axis. Lastly, it is important to highlight that a monoclonal antibody against IGF-1 receptor called teprotumumab has been developed for treatment of proptosis [37]. While hyperglycemia is a significant adverse effect, the existence of these agents offers the potential for their use in addressing DKD that involve excessive IGF activity.

This study had several limitations. First, biomarker data were unavailable for all participants; however, those in this post hoc analysis were similar to the main study. Second, more than 70% of study participants were White. A diverse research population can increase generalizability of our findings. Future studies need to implement the National Institutes of Health recommendations to conduct research studies with diverse ethnic backgrounds. Lastly, patients were followed up for 3 years; a longer duration may be required to investigate the association between IGF-1 and incident HF.

## Conclusion

In conclusion, this study provides evidence that elevated IGF-1 levels or the ratio of IGF-1/IGFBP-3 is associated with a higher risk of kidney disease progression and all-cause mortality. Three years of therapy with canagliflozin failed to impact IGF-1 or IGFBP-3 levels. Nonetheless, the benefit of canagliflozin to reduce cardio-renal endpoints was preserved across strata of both biomarkers. These results affirm a role of IGF-1 or its activity in the progression of cardio-renal disease among individuals with type 2 diabetes and DKD.

## Supplementary Information

**Additional file 1: Table S1.** Baseline characteristics according to IGFBP-3 quartiles. **Table S2.** Baseline characteristics according to IGF-1/IGFBP-3 quartiles. **Table S3.** Adjusted geometric mean ratio of biomarkers expressing adjusted relative difference in concentration at Year 1 and Year 3 versus baseline following treatment with either canagliflozin or placebo. **Table S4.** IGF-1 reference range. **Table S5.** Baseline characteristics of study population according to IGF-1 level. **Table S6.** Biomarker concentration across chronic kidney disease stages. **Figure S1.** CONSORT diagram. **Figure S2.** Restricted cubic spline model displaying the log hazard ratios for primary composite outcome.

## Data Availability

The data underlying this article cannot be shared publicly due to proprietary restrictions.

## Abbreviations

CV: Cardiovascular
CKD: Chronic kidney disease
DKD: Diabetic kidney disease
eGFR: Estimated glomerular filtration rate
GH: Growth hormone
HF: Heart failure
IGF-1: Insulin-like growth factor 1
IGFBP: Insulin-like growth factor binding protein
NT-proBNP: N terminal pro B type natriuretic peptides
UACR: Urine albumin creatinine ratio
