# Supplementary material for: Insulin growth factor axis and cardio-renal risk in diabetic kidney disease: an analysis from the CREDENCE trial
Source: Cardiovasc Diabetol. 2023 Jul 12;22:176. doi: 10.1186/s12933-023-01916-2 (PMC10339517; doi:10.1186/s12933-023-01916-2)
Supplement: Supplementary file 1 — Additional file 1: Table S1. Baseline characteristics according to IGFBP-3 quartiles. Table S2. Baseline characteristics according to IGF-1/IGFBP-3 quartiles. Table S3. Adjusted geometric mean ratio of biomarkers expressing adjusted relative difference in concentration at Year 1 and Year 3 versus baseline following treatment with either canagliflozin or placebo. Table S4. IGF-1 reference range. Table S5. Baseline characteristics of study population according to IGF-1 level. Table S6. Biomarker concentration across chronic kidney disease stages. Figure S1. CONSORT diagram. Figure S2. Restricted cubic spline model displaying the log hazard ratios for primary composite outcome. [file 12933_2023_1916_MOESM1_ESM.docx]

Additional file

**Insulin Growth Factor Axis and Cardio-renal Risk in Diabetic Kidney Disease: the CREDENCE Trial**

| Content | Page |
| --- | --- |
| Additional file 1: Table S1. Baseline characteristics according to IGFBP-3 quartiles. | 2-3 |
| Additional file 1: **Table S2**. Baseline characteristics according to IGF-1/IGFBP-3 quartiles. | 4-5 |
| Additional file 1: **Table S3**. Adjusted geometric mean ratio of biomarkers expressing adjusted relative difference in concentration at Year 1 and Year 3 versus baseline following treatment with either canagliflozin or placebo. | 6 |
| Additional file 1: **Table S4.** IGF-1 reference range. | 7 |
| Additional file 1: **Table S5**. Baseline characteristics of study population according to IGF-1 level. | 8-9 |
| Additional file 1: **Table S6**. Biomarker concentration across chronic kidney disease stages. | 10 |
| Additional file 1: **Figure S1**. CONSORT diagram. | 11 |
| Additional file 1: **Figure S2**. Restricted cubic spline model displaying the log hazard ratios for primary composite outcome. | 12-14 |

Additional file 1: **Table S1**. Baseline characteristics according to IGFBP-3 quartiles.

|  | **Q1 (N=644)** | **Q2 (N=644)** | **Q3 (N=644)** | **Q4 (N=643)** | *P* |
| --- | --- | --- | --- | --- | --- |
| **IGFBP-3, ng/mL** | 2050 (1744, 2263) | 2822 (2637, 3015) | 3607 (3428, 3834) | 4737 (4378, 5284) | <0.001 |
| **Canagliflozin, n (%)** | 330 (51.2) | 328 (50.9) | 318 (49.4) | 324 (50.4) | 0.92 |
| **Age, years, mean (SD)** | 66.40 (8.65) | 64.72 (8.55) | 62.90 (8.95) | 59.08 (8.51) | <0.001 |
| **Male, n (%)** | 491 (76.8) | 458 (71.6) | 433 (67.2) | 325 (50.6) | <0.001 |
| **Race, n (%)** |  |  |  |  | <0.001 |
| White | 496 (77.6) | 482 (75.3) | 441 (68.5) | 419 (65.3) |  |
| Asian | 54 (8.5) | 55 (8.6) | 99 (15.4) | 123 (19.2) |  |
| Black | 21 (3.3) | 38 (5.9) | 38 (5.9) | 44 (6.9) |  |
| **Comorbidities, n (%)** |  |  |  |  |  |
| Heart failure | 88 (13.7) | 80 (12.4) | 75 (11.6) | 88 (13.7) | 0.63 |
| Smoking | 89 (13.8) | 95 (14.8) | 85 (13.2) | 112 (17.4) | 0.15 |
| Hypertension | 625 (97.0) | 625 (97.0) | 616 (95.7) | 622 (96.7) | 0.46 |
| Coronary disease | 202 (31.4) | 194 (30.1) | 173 (26.9) | 162 (25.2) | 0.05 |
| Cerebrovascular disease | 100 (15.5) | 86 (13.4) | 108 (16.8) | 91 (14.2) | 0.33 |
| Peripheral artery disease | 192 (29.8) | 154 (23.9) | 162 (25.2) | 138 (21.5) | 0.006 |
| Chronic kidney disease | 599 (95.5) | 609 (96.2) | 598 (94.8) | 601 (95.1) | 0.64 |
| Obesity | 373 (58.1) | 373 (57.9) | 342 (53.3) | 392 (61.3) | 0.04 |
| eGFR, mL/min/1.73 m^2^ mean (SD) | 57.85 (18.11) | 57.17 (17.99) | 55.57 (18.40) | 56.16 (18.36) | 0.11 |
| Body mass index, kg/m^2^ mean (SD) | 32.11 (6.50) | 32.00 (6.07) | 31.07 (6.13) | 32.23 (6.27) | 0.003 |
| SBP, mmHg, mean (SD) | 142 (16) | 140 (16) | 140 (16) | 139 (15) | 0.01 |
| DBP, mmHg, mean (SD) | 77 (9) | 77 (9) | 78 (9) | 79 (10) | <0.001 |
| Hemoglobin A1c, mmol/mol , mean (SD) | 65 (13) | 66 (15) | 66 (14) | 68 (15) | 0.003 |
| LDL-C, mmol/L, Median (IQR) | 2.09 (1.56, 2.79) | 2.17 (1.63, 2.95) | 2.46 (1.78, 3.18) | 2.43 (1.81, 3.34) | <0.001 |
| HDL-C, mmol/L, Median (IQR) | 1.06 (0.88, 1.27) | 1.06 (0.91, 1.29) | 1.14 (0.96, 1.37) | 1.14 (0.93, 1.37) | <0.001 |
| Triglycerides, mg/dL Median (IQR) | 1.74 (1.26, 2.45) | 1.77 (1.29, 2.53) | 1.70 (1.26, 2.50) | 2.10 (1.52, 3.11) | <0.001 |
| **Diabetes duration, years, mean (SD)** | 17.42 (9.09) | 16.69 (8.89) | 16.55 (8.72) | 13.45 (7.52) | <0.001 |
| **Albumin creatinine ratio, mg/g, median (IQR)** | 98 (51, 197) | 100 (55, 179) | 105 (51, 217) | 105 (53, 206) | 0.75 |
| **Medications, n (%)** |  |  |  |  |  |
| Diuretic use | 337 (52.3) | 316 (49.1) | 318 (49.4) | 298 (46.3) | 0.20 |
| Statin use | 478 (74.2) | 478 (74.2) | 459 (71.3) | 437 (68.0) | 0.04 |
| Antithrombotic use | 448 (69.6) | 423 (65.7) | 406 (63.0) | 337 (52.4) | <0.001 |
| Beta blocker | 303 (47.0) | 264 (41.0) | 266 (41.3) | 262 (40.7) | 0.06 |
| Metformin | 370 (57.5) | 393 (61.0) | 396 (61.5) | 395 (61.4) | 0.39 |
| GLP-1 | 31 (4.8) | 40 (6.2) | 22 (3.4) | 34 (5.3) | 0.13 |
| Insulin | 459 (71.3) | 433 (67.2) | 418 (64.9) | 406 (63.1) | 0.01 |
| Sulfonylureas | 157 (24.4) | 171 (26.6) | 172 (26.7) | 188 (29.2) | 0.27 |
| **Biomarkers, median(IQR)** |  |  |  |  |  |
| NT-proBNP, pg/mL | 240 (105, 577) | 202 (93, 477) | 180 (81, 431) | 124 (58, 291) | <0.001 |
| Troponin T, ng/mL | 20 (14, 29) | 20 (13, 30) | 19 (12, 29) | 16 (11, 26) | <0.001 |
| IGF-1, ng/mL | 69 (53, 85) | 96.49 (78, 114) | 123 (98, 142) | 148 (117, 183) | <0.001 |
| IGF-1/IGFBP-3 × 1000 ratio | 34.62 (28.27, 41.76) | 33.87 (27.61, 40.24) | 34.16 (27.42, 39.55) | 30.46 (24.20, 37.57) | <0.001 |

eGFR: estimated glomerular filtration rate, SBP: systolic blood pressure, DBP: diastolic blood pressure, LDL-C: low-density lipoprotein cholesterol, HDL-C: high-density lipoprotein cholesterol, GLP-1: glucagon-like peptide 1, IQR: interquartile range, NT-proBNP: N terminal pro B type natriuretic peptides, IGF-1: insulin-like growth factor-1, IGFBP: insulin-like growth factor binding protein.

Additional file 1: **Table S2**. Baseline characteristics according to IGF-1/IGFBP-3 ratio quartile.

|  | **Q1 (N=642)** | **Q2 (N=641)** | **Q3 (N=641)** | **Q4 (N=641)** | *P* |
| --- | --- | --- | --- | --- | --- |
| IGF-1/IGFBP-3 × 1000 ratio | 22.66 (19.88, 24.77) | 30.15 (28.48, 31.71) | 36.40 (34.87, 38.16) | 45.53 (42.26, 50.28) | <0.001 |
| **Canagliflozin, n (%)** | 325 (50.6) | 345 (53.8) | 307 (47.9) | 316 (49.3) | 0.18 |
| **Age, years, mean (SD)** | 63.74 (8.64) | 63.44 (9.18) | 63.08 (9.15) | 62.79 (9.26) | 0.26 |
| **Male, n (%)** | 229 (35.7) | 411 (64.2) | 499 (78.3) | 562 (88.4) | <0.001 |
| **Race, n (%)** |  |  |  |  | 0.001 |
| White | 465 (72.4) | 447 (69.8) | 461 (72.4) | 457 (71.9) |  |
| Asian | 112 (17.4) | 88 (13.8) | 77 (12.1) | 53 (8.3) |  |
| Black | 20 (3.1) | 40 (6.2) | 37 (5.8) | 43 (6.8) |  |
| **Comorbidities, n (%)** |  |  |  |  |  |
| Heart failure | 101 (15.7) | 82 (12.8) | 83 (12.9) | 63 (9.8) | 0.02 |
| Smoking | 82 (12.8) | 107 (16.7) | 96 (15.0) | 95 (14.8) | 0.27 |
| Hypertension | 627 (97.7) | 616 (96.1) | 620 (96.7) | 615 (95.9) | 0.31 |
| Coronary disease | 182 (28.3) | 171 (26.7) | 175 (27.3) | 198 (30.9) | 0.35 |
| Cerebrovascular disease | 107 (16.7) | 81 (12.6) | 110 (17.2) | 85 (13.3) | 0.04 |
| Peripheral artery disease | 122 (19.0) | 152 (23.7) | 189 (29.5) | 179 (27.9) | <0.001 |
| Chronic kidney disease | 590 (93.9) | 594 (94.7) | 606 (95.7) | 607 (97.1) | 0.05 |
| Obesity | 381 (59.7) | 390 (61.0) | 354 (55.2) | 348 (54.4) | 0.04 |
| eGFR, mL/min/1.73 m^2^ mean (SD) | 59.26 (18.65) | 57.21 (18.06) | 56.93 (17.70) | 53.42 (18.11) | <0.001 |
| Body mass index, kg/m^2^ mean (SD) | 32.18 (6.51) | 32.14 (6.28) | 31.79 (6.33) | 31.30 (5.90) | 0.04 |
| SBP, mmHg, mean (SD) | 140 (15) | 140 (16) | 140 (16) | 140 (17) | 0.90 |
| DBP, mmHg, mean (SD) | 78 (9) | 78 (10) | 78 (10) | 78 (9) | 0.59 |
| Hemoglobin A1c, mmol/mol mean (SD) | 68 (15) | 68 (15) | 65 (13) | 64 (13) | <0.001 |
| LDL-C, mmol/L, Median (IQR) | 2.48 (1.81, 3.16) | 2.33 (1.76, 3.23) | 2.20 (1.63, 3.03) | 2.12 (1.60, 2.79) | <0.001 |
| HDL-C, mmol/L, Median (IQR) | 1.14 (0.96, 1.37) | 1.11 (0.93, 1.37) | 1.06 (0.91, 1.29) | 1.06 (0.88, 1.27) | <0.001 |
| Triglycerides, mmol/L, Median (IQR) | 190 (131, 275) | 164 (118, 240) | 155 (107, 224) | 150 (112, 200) | <0.001 |
| **Diabetes duration, years, mean (SD)** | 15.8 (8.9) | 16.3 (9.1) | 15.9 (8.2) | 16.2 (8.5) | 0.70 |
| **Albumin/creatinine ratio, mg/g, Median (IQR)** | 106 (52, 201) | 104 (51, 199) | 97 (52, 184) | 100 (55, 207) | 0.81 |
| **Medications, n (%)** |  |  |  |  |  |
| Diuretic use | 300 (46.7) | 317 (49.5) | 330 (51.5) | 317 (49.5) | 0.40 |
| Statin use | 445 (69.3) | 463 (72.2) | 461 (71.9) | 475 (74.1) | 0.30 |
| Antithrombotic | 375 (58.4) | 403 (62.9) | 411 (64.1) | 417 (65.1) | 0.07 |
| Beta blocker | 278 (43.3) | 257 (40.1) | 265 (41.3) | 292 (45.6) | 0.21 |
| Metformin | 407 (63.4) | 395 (61.6) | 393 (61.3) | 356 (55.5) | 0.03 |
| GLP-1 | 29 (4.5) | 43 (6.7) | 32 (5.0) | 23 (3.6) | 0.07 |
| Insulin | 420 (65.4) | 423 (66.0) | 434 (67.7) | 431 (67.2) | 0.81 |
| Sulfonylureas | 143 (22.3) | 176 (27.5) | 175 (27.3) | 193 (30.1) | 0.02 |
| **Biomarkers, median (IQR)** |  |  |  |  |  |
| NT-proBNP, pg/mL | 183 (74, 426) | 185 (81, 448) | 175 (84, 438) | 176 (82, 446) | 0.87 |
| Troponin T, ng/mL | 15 (10, 22) | 18 (12, 26) | 21 (13, 31) | 23 (16, 36) | <0.001 |
| IGF-1, ng/mL | 78 (56, 100) | 97 (74, 125) | 117 (92, 140) | 140 (105, 181) | <0.001 |
| IGFBP-3, ng/mL | 3473 (2628, 4468) | 3253 (2439, 4117) | 3188 (2529, 3846) | 2938 (2290, 3844) | <0.001 |

eGFR: estimated glomerular filtration rate, SBP: systolic blood pressure, DBP: diastolic blood pressure, LDL-C: low-density lipoprotein cholesterol, HDL-C: high-density lipoprotein cholesterol, GLP-1: glucagon-like peptide 1, NT-proBNP: N terminal pro B type natriuretic peptides, IGF-1: insulin-like growth factor-1, IGFBP: insulin-like growth factor binding protein.

Additional file 1: **Table S3**. Adjusted geometric mean ratio of biomarkers expressing adjusted relative difference in concentration at Year 1 and Year 3 versus baseline following treatment with either canagliflozin or placebo.

| **Biomarkers (log-transformed)** | **GMR (95% CI)** | *P* |
| --- | --- | --- |
| **Year 1** |  |  |
| IGF-1 | 1.03 (0.99, 1.07) | 0.07 |
| IGFBP-3 | 1.00 (0.98, 1.03) | 0.77 |
| IGF-1/IGFBP-3 × 1000 | 1.03 (1.00, 1.05) | 0.03 |
| **Year 3** |  |  |
| IGF-1 | 1.01 (0.95, 1.06) | 0.81 |
| IGFBP-3 | 1.01 (0.96, 1.05) | 0.77 |
| IGF-1/IGFBP-3 × 1000 | 1.00 (0.96, 1.04) | 0.94 |

Multivariable model adjusted for age, estimated glomerular filtration rate, body mass index, systolic blood pressure, hemoglobin A1c, duration of type 2 diabetes mellitus, UACR, history of heart failure, and current use of diuretic treatment

Additional file 1: **Table S4.** IGF-1 reference range.

| **Age, years** | **IGF-1 reference range, ng/mL** |
| --- | --- |
| 31-35 | 70-280 |
| 36-40 | 68-220 |
| 41-45 | 65-200 |
| 46-50 | 65-195 |
| 51-55 | 60-180 |
| 56-60 | 60-170 |
| 61-65 | 58-170 |
| 66-70 | 55-165 |
| ≥71 | 50-160 |

Additional file 1 **Table S5**. Baseline characteristics of study population according to IGF level.

|  | **Normal IGF-1** | **Elevated IGF-1** | *P* |
| --- | --- | --- | --- |
| n | 2299 | 270 |  |
| Age, years, mean (SD) | 63.59 (9.11) | 60.60 (8.12) | <0.001 |
| Male, n (%) | 1492 (65.2) | 210 (77.8) | <0.001 |
| **Race, n (%)** |  |  | 0.01 |
| White | 1655 (72.3) | 179 (66.3) |  |
| Asian | 295 (12.9) | 35 (13.0) |  |
| Black | 113 (4.9) | 27 (10.0) |  |
| **Comorbidities, n (%)** |  |  |  |
| Heart failure | 302 (13.1) | 29 (10.7) | 0.31 |
| Smoking | 341 (14.8) | 39 (14.4) | 0.94 |
| Hypertension | 2220 (96.6) | 262 (97.0) | 0.82 |
| Coronary disease | 658 (28.6) | 70 (25.9) | 0.39 |
| Cerebrovascular disease | 349 (15.2) | 35 (13.0) | 0.38 |
| Peripheral artery disease | 574 (25.0) | 69 (25.6) | 0.89 |
| Chronic kidney disease | 2144 (95.2) | 257 (96.6) | 0.39 |
| Obesity | 1319 (57.5) | 155 (57.4) | 1 |
| eGFR, mL/min/1.73 m^2^ | 57.26 (18.21) | 52.11 (17.91) | <0.001 |
| Body mass index, kg/m^2^ | 31.89 (6.31) | 31.56 (5.89) | 0.42 |
| SBP, mmHg | 140.19 (15.72) | 140.13 (16.26) | 0.96 |
| DBP, mmHg | 77.74 (9.47) | 78.60 (9.42) | 0.16 |
| Hemoglobin A1c, % | 8.25 (1.31) | 8.14 (1.25) | 0.16 |
| LDL-C, mg/dL | 88.00 (66.00, 119.00) | 85.00 (66.00, 115.00) | 0.48 |
| HDL-C, mg/dL | 43.00 (35.50, 51.00) | 42.00 (36.00, 51.00) | 0.40 |
| Triglycerides, mg/dL | 160.00 (115.00, 234.00) | 164.00 (125.00, 222.00) | 0.63 |
| Diabetes duration, years | 16.15 (8.76) | 14.89 (8.11) | 0.03 |
| Albumin/creatinine ratio, mg/g | 103.81 (52.10, 199.09) | 91.36 (52.00, 185.23) | 0.32 |
| **Medications, n (%)** |  |  |  |
| Diuretic use | 1123 (48.8) | 142 (52.6) | 0.27 |
| Statin use | 1654 (71.9) | 192 (71.1) | 0.83 |
| Antithrombotic use | 1453 (63.2) | 156 (57.8) | 0.09 |
| Beta blocker | 974 (42.4) | 119 (44.1) | 0.64 |
| Metformin | 1397 (60.8) | 157 (58.1) | 0.44 |
| GLP-1 | 116 (5.0) | 11 (4.1) | 0.58 |
| Insulin | 1532 (66.6) | 177 (65.6) | 0.77 |
| Sulfonylurea | 601 (26.1) | 86 (31.9) | 0.05 |
| **Biomarkers, median (IQR)** |  |  |  |
| NT-proBNP, ng/mL | 188.00 (83.70, 453.15) | 123.60 (63.40, 303.00) | <0.001 |
| Troponin T, ng/mL | 18.38 (12.24, 27.96) | 22.14 (15.42, 36.12) | <0.001 |
| IGF-1, ng/mL | 98.75 (74.44, 125.93) | 197.95 (180.60, 226.60) | <0.001 |
| IGFBP-3, ng/mL | 3055.36 (2385.48, 3857.97) | 4602.06 (3956.35, 5331.84) | <0.001 |
| IGF-1/IGFBP-3 × 1000 ratio | 22.66 (19.88, 24.77) | 30.15 (28.48, 31.71) | <0.001 |

eGFR: estimated glomerular filtration rate, SBP: systolic blood pressure, DBP: diastolic blood pressure, LDL-C: low-density lipoprotein cholesterol, HDL-C: high-density lipoprotein cholesterol, GLP-1: glucagon-like peptide 1, NT-proBNP: N terminal pro B type natriuretic peptides, IGF-1: insulin-like growth factor-1, IGFBP: insulin-like growth factor binding protein.

Additional file 1: **Table S6**. Median (interquartile range) of biomarker levels across chronic kidney disease stages.

|  | **Stage 1**  eGFR≥ 90 | **Mild 2**  eGFR: 60-90 | **Moderate 3**  eGFR: 30-59 | **Severe 4**  eGFR: 15-29 | *P* |
| --- | --- | --- | --- | --- | --- |
| n | 139 | 945 | 1433 | 110 |  |
| eGFR, mL/min/1.73 m^2^ | 94 (92, 98) | 71 (65, 78) | 45 (38, 53) | 27 (25, 28) | <0.001 |
| IGF-1, ng/mL | 95 (70, 137) | 102 (76, 132) | 106 (79, 139) | 116 (84, 151) | 0.002 |
| IGFBP-3, ng/mL | 3196 (2398, 4109) | 3171 (2428, 4034) | 3227 (2470, 4095) | 3580 (2509, 4368) | 0.24 |
| IGF-1/IGFBP-3 ratio × 1000 | 31 (24, 39) | 33 (26, 39) | 34 (27, 41) | 35 (29, 43) | 0.001 |

eGFR: estimated glomerular filtration rate, IGF-1: insulin-like growth factor-1, IGFBP: insulin-like growth factor binding protein.

Additional file 1: **Figure S1**: Consort diagram.


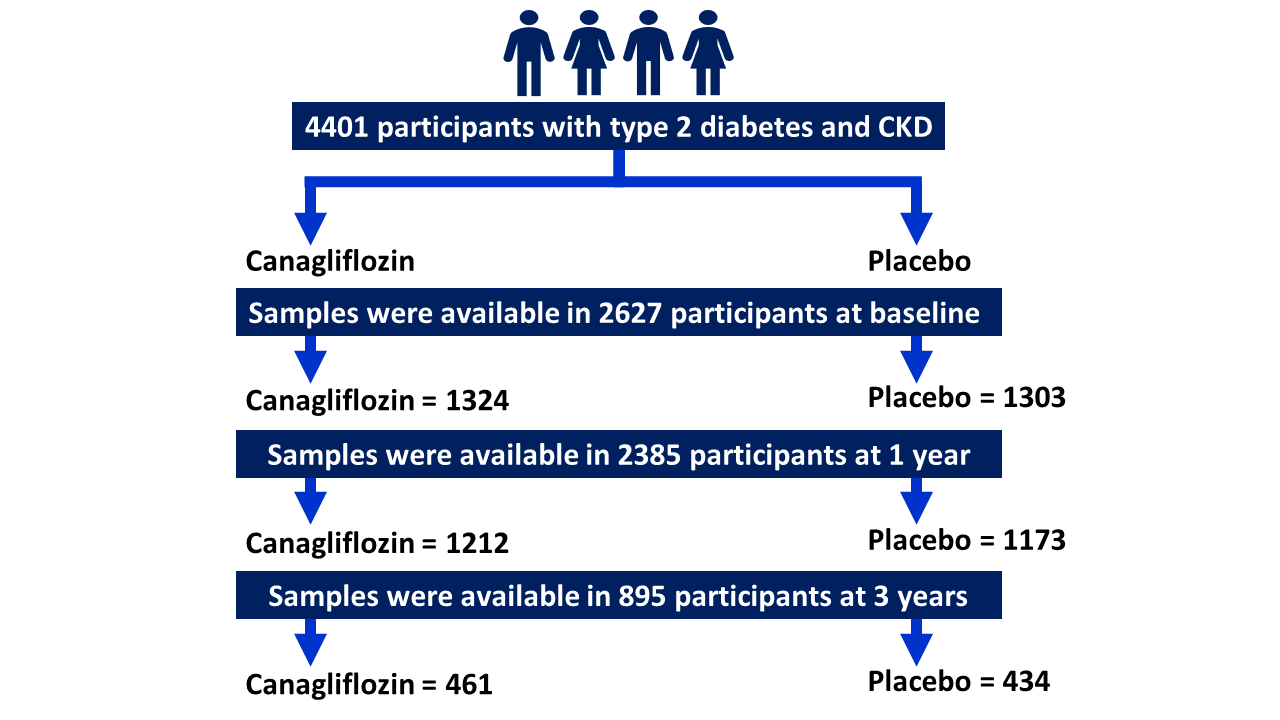


CKD: chronic kidney disease.

Additional file 1: **Figure S2**. Restricted cubic spline model displaying the log hazard ratios for primary composite outcome by A) IGF-1 level, B) IGFBP-3 and C) IGF-1/IGFBP ratio.


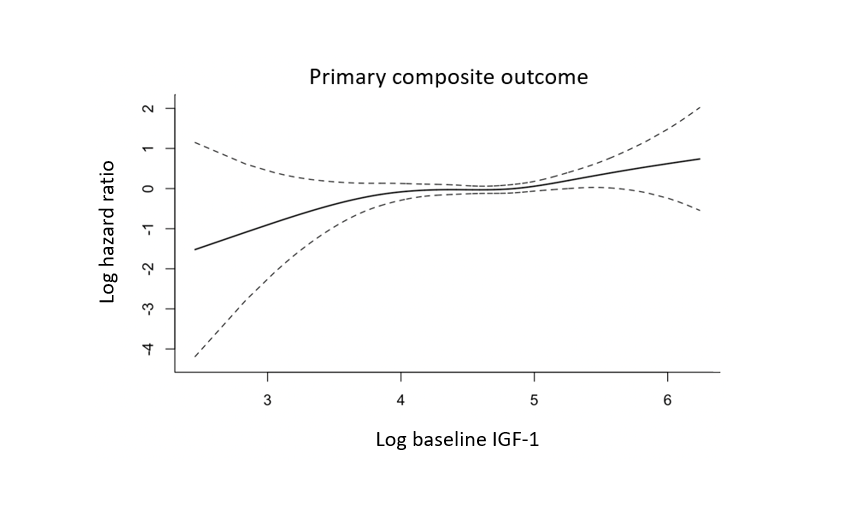


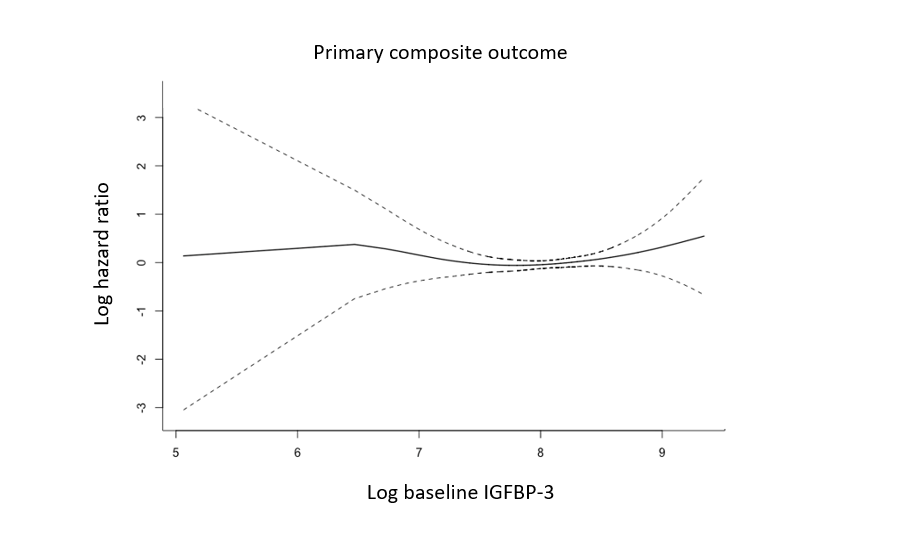


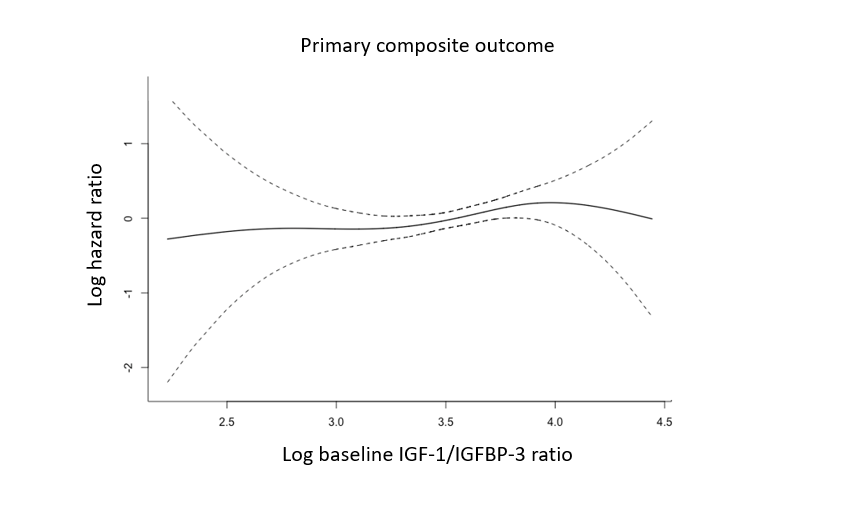


IGF: insulin-like growth factor, IGFBP: insulin growth factor binding protein.
